# Phase I and II Clinical Trial Comparing the LBSap, Leishmune^®^, and Leish-Tec^®^ Vaccines against Canine Visceral Leishmaniasis

**DOI:** 10.3390/vaccines8040690

**Published:** 2020-11-17

**Authors:** Rodrigo Dian de Oliveira Aguiar-Soares, Bruno Mendes Roatt, Fernando Augusto Siqueira Mathias, Levi Eduardo Soares Reis, Jamille Mirelle de Oliveira Cardoso, Rory Cristiane Fortes de Brito, Henrique Gama Ker, Rodrigo Corrêa-Oliveira, Rodolfo Cordeiro Giunchetti, Alexandre Barbosa Reis

**Affiliations:** 1 Laboratório de Imunopatologia, Núcleo de Pesquisas em Ciências Biológicas/NUPEB, Universidade Federal de Ouro Preto, CEP 35400-000 Ouro Preto, Brazil; rodrigo.soares@ufop.edu.br (R.D.d.O.A.-S.); roatt@ufop.edu.br (B.M.R.); fa_mathias@yahoo.com.br (F.A.S.M.); levieduardosreis@gmail.com (L.E.S.R.); ja_mirelle@yahoo.com.br (J.M.d.O.C.); rorybrito@gmail.com (R.C.F.d.B.); hgker@hotmail.com (H.G.K.); 2 Departamento de Análises Clínicas, Escola de Farmácia, Universidade Federal de Ouro Preto, CEP 35400-000 Ouro Preto, Brazil; 3 Instituto Nacional de Ciência e Tecnologia em Doenças Tropicais (INCT-DT), CEP 40110-040 Salvador, Brazil; 4 Laboratório de Imunologia Celular e Molecular, Centro de Pesquisas René Rachou, Fundação Oswaldo Cruz-FIOCRUZ, CEP 30190-009 Belo Horizonte, Brazil; correa@cpqrr.fiocruz.br; 5 Laboratório de Biologia das Interações Celulares, Departamento de Morfologia, Universidade Federal de Minas Gerais, CEP 31270-901 Belo Horizonte, Brazil; giunchetti@gmail.com

**Keywords:** visceral leishmaniasis, vaccines, LBSap, Leishmune^®^, Leish-Tec^®^, *Leishmania infantum*, dog

## Abstract

In this study, we performed a phase I and II clinical trial in dogs to evaluate the toxicity and immunogenicity of LBSap-vaccine prototype, in comparison to Leishmune^®^ and Leish-Tec^®^ vaccines. Twenty-eight dogs were classified in four groups: (i) control group received 1 mL of sterile 0.9% saline solution; (ii) LBSap group received 600 μg of *Leishmania braziliensis* promastigotes protein and 1 mg of saponin adjuvant; (iii) Leishmune^®^; and (iv) Leish-Tec^®^. The safety and toxicity of the vaccines were measured before and after three immunizations by clinical, biochemical, and hematological parameters. The clinical examinations revealed that some dogs of LBSap and Leishmune^®^ groups presented changes at the site of vaccination inoculum, such as nodules, mild edema, and local pain, which were transient and disappeared seventy-two hours after vaccination, but these results indicate that adverse changes caused by the immunizations are tolerable. The immunogenicity results demonstrate an increase of B lymphocytes CD21^+^ regarding the Leishmune^®^ group and monocytes CD14^+^ concerning LBSap and Leishmune^®^ groups. In the in vitro analyses, an increase in lymphoproliferative activity in LBSap and Leishmune^®^ groups was observed, with an increase of antigen-specific CD4^+^ and CD8^+^ T lymphocytes in the LBSap group. A second approach of in vitro assays aimed at evaluating the percentage of antigen-specific CD4^+^ and CD8^+^ T lymphocytes producers of IFN-γ and IL-4, where an increase in both IFN-γ producing subpopulations in the LBSap group was observed, also showed an increase in IFN-γ producers in CD8^+^ lymphocytes in the Leish-Tec^®^ group. Our data regarding immunogenicity indicate that the vaccination process, especially with the LBSap vaccine, generated a protective immune response compatible with *L. infantum* parasite control. Based on the foregoing, the LBSap vaccine would be suitable for further studies of phase III clinical trial in endemic areas with high prevalence and incidence of canine visceral leishmaniasis (VL) cases.

## 1. Introduction

Visceral leishmaniasis (VL) is endemic in 88 countries in the world with approximately 90% of reported cases concentrated in India, Sudan, South Sudan, Bangladesh, Ethiopia, and Brazil [1,2]. In the Americas, VL is present in 12 countries [3], with 96% of the cases being reported in Brazil [2]. From 1990 to 2016, 84,922 cases of VL were confirmed in Brazil, with the case-fatality rate reaching 7.4% in 2016 [4,5]. In an attempt to establish control measures, the Ministry of Health in Brazil advocates, as an epidemiological practice, the treatment of sick people, insecticide sprays with residual effect at home and peridomicile against the vector, as well as the euthanasia of the urban reservoir of the parasite, the dog [6,7]. However, the euthanasia of seropositive dogs, often without any clinical signs of infection, becomes increasingly questionable and unsustainable [8,9,10]. Unfortunately, to date, there is no effective therapeutic regimen to promote parasitological cure in canine visceral leishmaniasis (CVL), keeping these animals as reservoir of the parasite and maintaining transmission to sandflies [11,12]. In addition, the treatment of CVL with leishmanicidal drugs used in humans may lead to the selection of drug-resistant strains and, in many countries, this therapy has been contraindicated or prohibited [6].

Thus, immunoprophylaxis against CVL could provide long-lasting and specific immunity against the *Leishmania infantum* parasite, preventing the infection of dogs. Moreover, taking together their cost/benefit ratio and their easy use, prophylactic vaccines are the most effective prevention and control instruments at our disposal to be considered to control canine infection and prevent human infection [1,13,14,15]. In addition, vaccines could also function as important tools for the treatment of VL dogs, creating a double perspective in the application of this strategy in endemic countries [16,17].

Different studies have been conducted in an attempt to better understand the course of the infection and to identify biomarkers of resistance and susceptibility that can guide the development and testing of anti-CVL immunobiologicals [13,18]. Thus, among the resistance biomarkers in CVL, the following stand out: (i) the predominant presence of IgG1 associated with a lower frequency of parasitic intensity in several tissues [19]; (ii) increase in total circulating T lymphocytes and their subpopulations (CD4^+^ and CD8^+^) in peripheral blood [20]; (iii) increase in total anti-*Leishmania* T splenocytes and their subpopulations (CD4^+^ and CD8^+^), mainly T (CD8^+^) [21]; (iv) mild or moderate histopathologic changes in the skin, liver, spleen, and popliteal lymph node, often accompanied by a lower parasitic burden; and (v) mixed cytokine profile, with high preferential IFN-γ production, TNF-α, and IL-13 [18,22]. Considering the current lack of well-standardized studies and which methodologies should be used, it is necessary to carry out comparative studies using the same conditions, to better determine the protection of potential vaccines against CVL in a single clinical trial [13].

The glycoprotein preparation enriched with *L. donovani* promastigotes, named FML (fucose-mannose ligand), antigen for human use [23] and canine [24], was formulated with the adjuvant *Quillaja saponaria* saponin and underwent phase I, II, and III trials becoming the first licensed CVL vaccine in Brazil under the name of Leishmune^®^ (Zoetis Industria de Produtos Veterinarios LTDA, São Paulo, Brazil) [25]. In vaccine phase III field trials with dogs, the vaccine demonstrated 92% to 95% protection against CVL in the vaccinated group corresponding to 76% vaccine efficacy [26,27]. In 2014, MAPA/Brazil suspended the license to manufacture and commercialize the Leishmune^®^ vaccine [28], because the company did not comply with the technical regulation approved in the Interministerial Normative Instruction 31 (IN-31/2007) [29].

Currently, the only vaccine available in the Brazilian market for immunization against CVL is Leish-Tec^®^ (Hertape Calier Saúde Animal S/A, Juatuba, Brazil), composed of recombinant protein A2 associated with the adjuvant saponin [30]. In phase III vaccine trials, efficacy reached 80.8% in which xenodiagnosis detected a 46.6% reduction in transmission to sandflies in vaccinated dogs [31]; however, Grimaldi et al. [32] estimate that immunoprophylaxis by the Leish-Tec^®^ vaccine may not have an impact on reducing the incidence of CVL in endemic areas with high rates of disease transmission.

Our research group studied a vaccine composed of crude antigens of *L. braziliensis* promastigotes associated with saponin adjuvant (LBSap) [33,34,35]. The LBSap vaccine demonstrated strong immunogenicity both within the cellular and humoral immune response, indicating the establishment of immunoprotective mechanisms potentially capable of acting against infection by the etiological agent of CVL [33,36], and more recently, we demonstrated that this vaccine can have an immunotherapeutic use in CVL [17].

Considering these promising results demonstrated by the LBSap vaccine and the need to better understand the aspects of the immune response, we propose in the present study to extend the phase I and II clinical trial evaluations in immunized dogs with the LBSap vaccine, using a detailed analysis of the safety and immunogenicity after three doses, parallelly compared with two CVL vaccines, the Leishmune^®^ vaccine produced by Zoetis (São Paulo, Brazil) which was commercially available in Brazil until 2014 and the Leish-Tec^®^ vaccine produced by Hertape Calier Saúde Animal (Juatuba, Brazil) which is available in clinics for use in dogs in Brazil.

## 2. Materials and Methods

The study protocol number 2010/71 was approved by the Ethical Committee for the Use of Experimental Animals at the Federal University of Ouro Preto, Minas Gerais, Brazil.

### 2.1. Study Animals and Vaccination Protocol

We used twenty-eight male and female mongrel dogs that were born and reared in the kennels of the Animal Science Center, Federal University of Ouro Preto, Ouro Preto, Minas Gerais, Brazil. The dogs were treated until 7 months of age with anthelmintic and vaccinated against rabies (Tecpar, Curitiba, Brazil), canine distemper, type 2 adenovirus, coronavirus, parainfluenza, parvovirus, and leptospira (Vanguard^®^ HTLP 5/CV-L; Pfizer Animal Health, New York, NY, USA). The absence of specific anti-*Leishmania* antibodies was confirmed by rapid immunochromatographic Dual-Path Platform technology (DPP^®^—Bio-Manguinhos^®^, Manguinhos, Brazil) and immunoenzymatic assay (EIE^®^—Bio-Manguinhos^®^, Manguinhos, Brazil) tests. Animals were divided in four experimental groups: (i) the Control (C) group (*n* = 7) that received 1 mL of sterile 0.9% saline; (ii) the LBSap group (*n* = 7) that received 600 µg of *L. braziliensis* promastigote protein plus 1 mg of saponin in 1 mL of sterile 0.9% saline; (iii) the Leishmune^®^ group (*n* = 7) that received the vaccine Leishmune^®^ according to the manufacturer’s recommendation; (iv) the Leish-Tec^®^ group (*n* = 7) that received the commercial vaccine Leish-Tec^®^, according to the manufacturer’s recommendation. Each group received three subcutaneous injections in the right flank at intervals of 21 days.

### 2.2. Blood Sample Collection

We collected the peripheral blood from the dogs before the vaccination started (day 0 = T0 = before vaccination) and 15 days after completing the vaccination protocol (day 78 = T1 = after vaccination). For immunophenotyping, hematology, and biochemistry exams, blood was collected as described by [35] and stored at room temperature.

### 2.3. Local and Systemic Reactions to Immunization

During the three immunizations protocols, a detailed toxicity/safety study was carried out using a rigorous clinical follow-up through the daily evaluation of macroscopic changes at the immunization site and behavior of the dogs. Local tolerance was determined by direct visual examination, and when lesions were observed, they were measured at 24 h intervals over a period of 72 h after each immunization. In addition, temperatures, weights, and biochemical profile were measured to establish an overall evaluation of the animal’s health.

Biochemical examinations consisted of assessing renal function by measuring creatinine and urea. For the assessment of liver function, the enzymes were dosed: alkaline phosphatase (ALP), aspartate aminotransferase (AST/GOT), alanine transaminase (ALT/GPT), gamma-glutamyltransferase (γ-GT), and bilirubin. In addition, we performed the proteinogram tests through the measurements of albumin, globulin, albumin/globulin ratio, and total protein. All biochemical evaluations of the study were carried out in the Automatic Biochemical System (CELM SBA-200, Barueri, Brazil) using commercial kits from Labtest (Labtest Diagnostica S.A., Lagoa Santa, Brazil), according to the manufacturer’s recommendations. With the intention of creating a normal reference range for biochemical assessments, blood was collected from forty-five healthy dogs born and raised in the kennel of the Animal Science Center of the Federal University of Ouro Preto.

### 2.4. Humoral Immune Response: Anti-Leishmania Antibody Test

Serum samples from the different groups of dogs were submitted to the DPP^®^ and to the EIE^®^, according to the manufacturer’s recommendations. For the DPP^®^, after the addition of the sera sample and the application of running buffers, the reading was performed after 15 min and the results were considered positive when two lines were visualized. The negative/positive discriminant cut-off point of the EIE^®^ was determined according to label recommendations. Thus, the negative/positive discriminant cut-off point, evaluated by optical density, was obtained for the reactions performed by the comparative analysis between the groups Control, LBSap, Leishmune^®^, and Leish-Tec^®^ (Cut-off: 0.173). The plates were read on a Multiskan^®^ MCC 340 microplate reader (Labsystems, Helsinki, Finland) at 450 nm absorbance. The results were expressed by the mean optical density of each group after the complete vaccination protocol.

### 2.5. Hematological Analysis and Ex Vivo Immunophenotyping

The BC-2800 VET automatic hematology analyzer (Mindray, Guangdong, China) was used to obtain the erythrogram values and the total circulating leukocyte count. To perform the differential leukocyte count, blood smear slides from each animal were made. To obtain normal reference values for hematological parameters, forty-five control dogs born and bred in the kennel of the Animal Science Center of the Federal University of Ouro Preto were used. The leukocyte immunophenotyping assays were conducted, with some modifications, according to De Almeida et al. [37]. Canine monoclonal antibodies (mAbs) purchased from Serotec, USA, were used in an immunofluorescence procedure by flow cytometry: anti-CD3 PE (clone CA17.2A12), anti-CD4 FITC (clone YKIX302.9), anti-CD8 AF647 (clone YCATE55.9), anti-CD21 PE (clone CA2.1D6), anti-CD14 RPE-Cy5 (TÜK4), anti-CD5 PE (clone YKIX322.3), anti-human-CD16 APC (3G8), and anti-human-CD25 PE (clone P4A10; purchased from eBioscience). The results were expressed according to Roatt and colleagues [17] in absolute counts (cell number per cubic meter) through the product of the percentage of positive cells (CD3^+^, CD8^+^, CD4^+^, CD21^+^, and CD5^−^CD16^+^) within gated lymphocytes by absolute lymphocyte counts. The absolute counts for monocytes were obtained by selecting the specific region of interest in graphics, based on the specific labeling for CD14^+^ associated with the distribution of the granularity of the cells. The results of T lymphocytes expressing interleukin-2 receptor alpha chain (CD25^+^) were demonstrated in the percentage of CD4^+^CD25^+^ or CD8^+^CD25^+^ T lymphocytes within the total CD4^+^ or CD8^+^ T lymphocytes, respectively. Acquisitions were performed in a FACSCalibur (Becton Dickinson, San Jose, CA, USA) flow cytometer in total of 20,000 events and the data were analyzed using FlowJo software (Becton, Dickinson and Company, Franklin Lakes, NJ, USA).

### 2.6. In Vitro Proliferation Assays

In our study, an in vitro proliferation assay was performed, using *L. infantum* soluble antigen (SLiA) [19], according to the protocol, with some modifications, described by Roatt and colleagues [17]. First, 20 mL of heparinized blood were collected from the animals and applied in 30 mL of Ficoll-Hypaque density gradient (Histopaque^®^ 1.077; Sigma Chemical Co., St. Louis, MO, USA) for subsequent centrifugation at 450× *g* for 40 min, at room temperature in order to obtain peripheral blood mononuclear cells (PBMCs). Briefly, PBMCs were washed three times in RPMI 1640, followed by counting in a Neubauer chamber and adjusting the final concentration to 1 × 10^7^ cells/mL. The PBMCs were incubated at 37 °C, for 10 min, for labeling with the succinimidyl ester of fluorescein carboxy diacetate (CFSE, Molecular Probes, Eugene, OR, USA) at a final concentration of 10 µM. Right after that, we added 5 mL of ice-cold RPMI 1640 plus 10% FBS and the tubes with the marked PBMCs were placed in ice for 5 min, after which they were washed two times with PBS at 300× *g* for 7 min. Once marked with CFSE, PBMCs (50 μL–5 × 10^5^) were plated in the wells of 48-well flat-bottom plates, suitable for tissue culture (Costar, Cambridge, MA, USA). In each of the wells containing the PBMCs, 650 μL of supplemented RPMI medium were added for further incubation with 5% CO_2_ at 37 °C for 5 days. SLiA (25 μg/mL) was added to the wells for specific stimulation or RPMI 1640 as a control. In addition, PBMCs were also grown separately in wells for positive control in mitogenic stimulus assays in which 25 μL of phytohemagglutinin (2.5 μg/mL; Sigma-Aldrich Chemie GmbH, Taufkirchen, Germany) was added. Subsequently, after 5 days of incubation, the culture supernatant was collected and stored in a −80 °C freezer. Then, the PBMCs were removed from the culture plates and transferred to tubes, where they were washed twice with PBS and stained at room temperature for 30 min with anti-CD8 AF647 (clone YCATE55.9) and anti-CD4 PE (clone YKIX302.9), purchased from Serotec, USA. Subsequently, PBMCs were washed with PBS, centrifuged at 300× *g* for 10 min, resuspended, and fixed. The proliferation assay was read on the flow cytometer (FACScalibur—Becton Dickinson, San Jose, CA, USA) with a total of 50,000 events per tube. The final data for the total and specific subset of T lymphocytes proliferation were expressed as indexes. The index was calculated by dividing the percentage of positive cells observed in the SLiA-stimulated culture by the one observed in paired control unstimulated culture (SLiA/CC ratio).

### 2.7. TNF-α and IL-10 Quantification

The supernatant that was collected in the in vitro PBMC proliferation assay was used for the quantification of cytokines by the capture ELISA technique, according to Roatt and collaborators [17]. For this, we used the following antibodies from the company R&D Systems (Minneapolis, MN, USA): mouse monoclonal anti-canine IL-10 and TNF-α antibodies (mAb) and biotinylated polyclonal goat anti-canine IL-10 and TNF-α (R&D Systems, Minneapolis, MN, USA). Briefly, we used ninety-six well plates and 1.0 μg/mL of mAb was added to capture the respective cytokines in the samples, followed by the application of the respective detection antibodies (at a concentration of 1.5 μg/mL) and later reaction with 3,3′,5,5′-tetramethylbenzidine (Sigma-Aldrich, St. Louis, MO, USA). Standard curves were made using TNF-α and IL-10 (R&D Systems, Minneapolis, MN, USA), following the kit’s recommendations. The automatic microplate reader EL 800G ELISA (Bio Tek Instruments, Winooski, VT, USA) with 450 nm filter was used to read the plates. The values of TNF-α and IL-10 cytokines detected in the supernatant were expressed as indexes, calculated by dividing the cytokine concentration (pg/mL) observed in the SLiA-stimulated culture by the one observed in paired control unstimulated culture (SLiA/CC ratio).

### 2.8. In Vitro Intracellular Cytokines Assays

In vitro assays for labeling intracytoplasmic cytokines were conducted as previously published by Roatt and colleagues [17], which consisted primarily of immunostaining cell surface markers, followed by the assay for intracellular cytokine labeling. Briefly, three polypropylene tubes were used in the experiment for each dog in which 1 mL of whole blood in heparin was added to 1 mL of RPMI in each tube for incubation in a humidified atmosphere with 5% CO_2_ at 37 °C for a period of 12 h. The first tube consisted of a control tube (blood and RPMI only). The second tube consisted of the one stimulated with SLiA (25 μg/mL). The third tube was the positive control. After the first 12 h of incubation, Brefeldin A (BFA) was added in a final concentration of 10 μg/mL (Sigma, St. Louis, MO, USA) in all tubes, for subsequent incubation, under the same previous conditions, for another 4 h. PMA (25 ng/mL) was added to the positive control tube with BFA followed by the same 4 h of culture. Erythrocytes were lysed and then the first staining was performed using monoclonal anti-surface molecules (anti-CD4 FITC—clone YKIX302.9 and anti-CD8 AF647—clone YCATE55.9; Serotec Ltd., Oxford, England). After the cell permeabilization, we proceeded to stain intracytoplasmic cytokines using anti-IFN-γ (clone CC302) and anti-IL-4 (clone CC303) (Serotec Ltd., Oxford, UK) in U-bottom 96-well plates. The samples were read on the FACSCalibur flow cytometer (Becton Dickinson, San Jose, CA, USA) in which 30,000 events were acquired per well. Final data of intracytoplasmic cytokine production assay were expressed as indexes, calculated by dividing the percentage of positive cells observed in the SLiA-stimulated culture by the one observed in paired control unstimulated culture (SLiA/CC ratio).

### 2.9. Statistical Analysis

Prism 6.0 software package was used to perform the statistical analyses (Prism Software, Irvine, CA, USA). To confirm the normality of the data, a Kolmogorov–Smirnov test was used for immunophenotypic profiles (*ex vivo*), proliferation response, supernatant, and intracytoplasmic cytokines production. Data were analyzed for statistical differences using one-way ANOVA test followed by Dunnett’s multiple comparison tests. Analyses comparing the times before and after vaccination in the same group were performed using paired *t*-test. Differences were considered significant when *p*-values were <0.05.

## 3. Results

### 3.1. Changes Observed at the Site of Vaccination Inoculum Were Transient and Quickly Disappeared, Indicating that Adverse Changes Caused by the Immunizations Are Tolerable in All Vaccines Tested

The main macroscopic changes observed at the inoculum site during vaccination were small nodules, non-ulcerated, firm consistency, with variable dimensions (0.1 to 1 cm^2^), almost unnoticeable, disappearing after 72 h of vaccination [LBSap (7/7) and Leishmune^®^ (5/6)] (Supplementary Table S1). The number of dogs with changes at the inoculum site increases according to subsequent vaccinations. Dogs with local changes in the first dose also had nodules/edema in subsequent doses. Four dogs in the LBSap group and three dogs in the Leishmune^®^ group presented nodules/edema only with the third dose of the vaccine. A dog in each of the vaccine groups LBSap and Leishmune^®^, after the third vaccine dose, presented palpation pain at the inoculation site of the vaccine, which disappeared after 72 h (Supplementary Table S1). Rectal temperature measurements of dogs were also performed at 24, 48, and 72 h after each of the three vaccine doses, and most dogs from the different groups evaluated maintained body temperature within the normal range (up to 39.5 °C) for canines (Supplementary Table S1). In all clinical observations after each dose of the different vaccination protocols, no toxicological, physiological, and behavioral changes such as: vomiting, diarrhea, tremors, weight loss, and apathy were detected. No changes were found in the hematological and biochemical parameters (leukogram, erythrogram, liver function, renal function, and proteinogram) of the dogs at the end of the vaccine protocol evaluated (Supplementary Tables S2–S5).

### 3.2. LBSap and Leishmune^®^ Vaccines Elicited an Intense Immunogenic Reaction that Was Characterized by Elevated Levels of IgG-Total Antibodies to Leishmania

**Figure 1:**
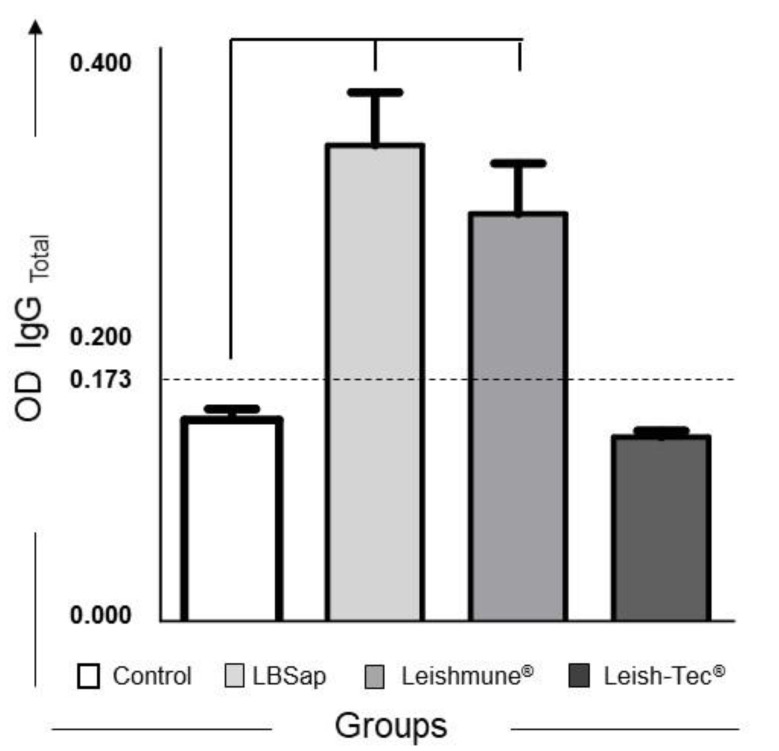
Anti-*Leishmania* total IgG reactivity in serum from dogs submitted to different vaccination protocols by immunoenzymatic assay (EIE^®^—Bio-Manguinhos^®^). The *x*-axis displays the vaccine groups: C (control; 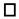); LBSap (killed *L. braziliensis* vaccine plus saponin; 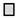); Leishmune^®^ (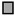); Leish-Tec^®^ (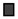). The *y*-axis represents the mean ELISA absorbance values determined at 450 nm in serum samples for total IgG. The cut-off is represented by the dotted line. Significant differences (*p* < 0.05) between the C group and the LBSap, Leishmune^®^, and Leish-Tec^®^ groups are indicated by connecting lines.

Figure 1 shows the results of serum IgG-Total reactivity evaluation by the EIE^®^ in vaccinated groups. We observed a significant increase (*p* < 0.05) in the mean of the optical density values after vaccination in the LBSap (T1: 0.249 ± 0.073) and Leishmune^®^ (T1: 0.213 ± 0.065) in relation to the Control (T1: 0.106 ± 0.015) group (Figure 1)). Analyzing the reactivity of vaccinated animals using the DPP^®^, we observed no positive dogs among all dogs studied.

### 3.3. LBSap and Leishmune^®^ Vaccines Elicited an Increase in the Numbers of Circulating CD14^+^ Monocytes

**Figure 2:**
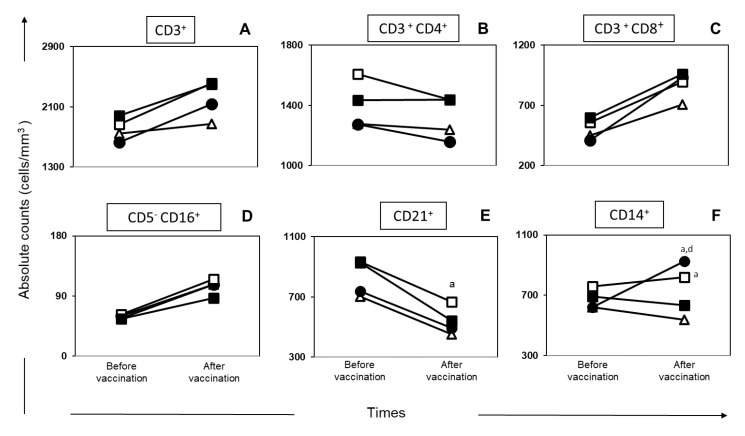
Cellular profile of circulating leukocytes in dogs submitted to different vaccination protocols. C (control; 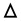); LBSap (killed *Leishmania braziliensis* vaccine plus saponin; 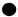) Leishmune^®^ (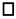); Leish-Tec^®^ (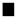). The *x*-axis displays the times of the assays (time before vaccination and time after vaccination), and the *y*-axis represents the mean values of absolute counts: (**A**) CD3^+^ lymphocytes; (**B**) CD3^+^CD4^+^ lymphocytes; (**C**) CD3^+^CD8^+^ lymphocytes; (**D**) CD5^−^CD16^+^ natural killer cells; (**E**) CD21^+^ B cells; (**F**) CD14^+^ monocytes. Significant differences (*p* < 0.05) in relation to the C or Leish-Tec® groups are represented by the letters a and d, respectively.

In order to evaluate the immunophenotypic profile of the vaccinated dogs, we evaluated the frequency of T lymphocytes (CD3^+^), their major subpopulations (CD4^+^ and CD8^+^) and NK cells (CD5^−^CD16^+^) in peripheral blood (Figure 2A–D). Our results revealed no changes (*p* < 0.05) in the number of this circulating cells in dogs immunized by any of the vaccines tested. However, we observed an increase (*p* < 0.05) in the number of circulating CD21^+^ B lymphocytes in dogs of Leishmune^®^ after vaccination when compared with the Control group (Figure 2E). In addition, we observed a relevant increase (*p* < 0.05) in CD14^+^ monocytes after vaccination in Leishmune^®^ dogs compared with the Control group (Figure 2F). Similarly, the LBSap group showed a significant increase (*p* < 0.05) in CD14^+^ monocytes after vaccination compared with the Control and Leish-Tec^®^ groups (Figure 2F).

**Figure 3:**
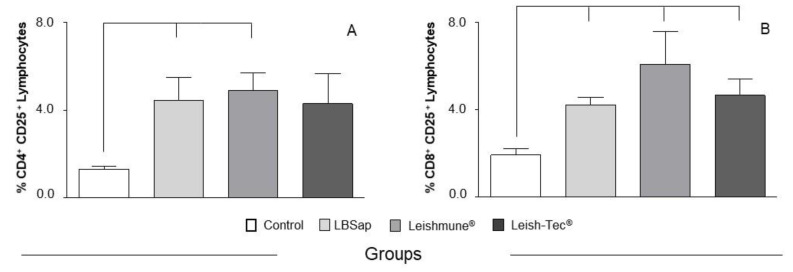
Cellular profile of circulating regulatory T lymphocytes in dogs submitted to different vaccination protocols. The *x*-axis displays the groups: C (control; 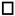); LBSap (killed *L. braziliensis* vaccine plus saponin; 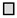); Leishmune^®^ (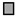); Leish-Tec^®^ (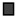). The *y*-axis represents the mean values of percentage counts of (**A**) CD4^+^CD25^+^ lymphocytes and (**B**) CD8^+^CD25^+^ lymphocytes at the time after vaccination. Significant differences (*p* < 0.05) between the C group and the LBSap, Leishmune^®^, and Leish-Tec^®^ groups are indicated by connecting lines.

Evaluating the activated cells, we observed, after the complete vaccination protocol, an increase (*p* < 0.05) in the percentage of CD4^+^ T lymphocytes expressing the CD25^+^ marker in LBSap and Leishmune^®^ groups in relation to the Control group (Figure 3A). However, we observed an increase (*p* < 0.05) in the percentage of CD8^+^ CD25^+^ T cell population in all vaccine groups (LBSap, Leishmune^®^, and Leish-Tec^®^) in relation to the Control group (Figure 3B).

### 3.4. In Vitro Cell Proliferation in the Presence of Antigenic Stimuli Was Intensely Increased Following Vaccination with LBSap and Leishmune^®^, with an Increase of Antigen-Specific CD4^+^ and CD8^+^ T Lymphocytes and Secretion of TNF-α Cytokines Only in LBSap Group

**Figure 4:**
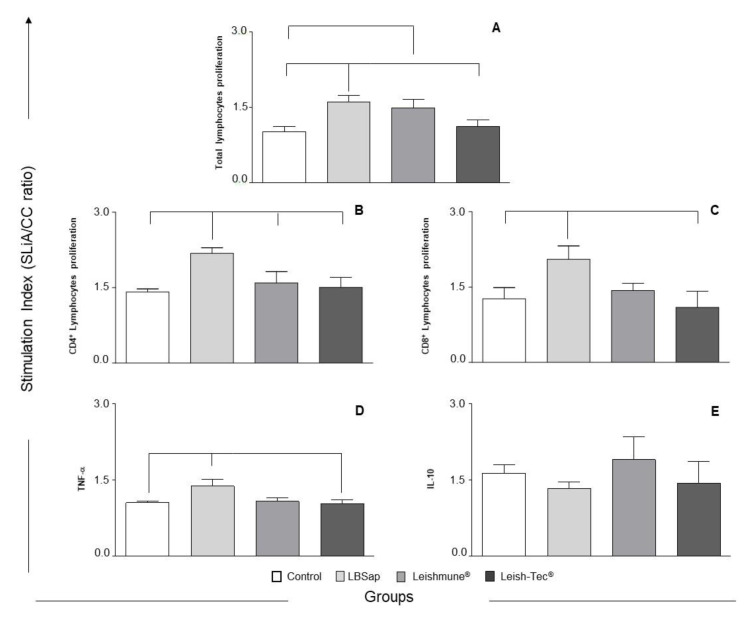
Cell proliferation response of peripheral blood mononuclear cells (PBMCs) after stimulation with soluble *L. infantum* antigen (SLiA) and profile of cytokine production in the culture supernatant of dogs submitted to different vaccination protocols. The *x*-axis displays the groups: C (control; 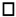); LBSap (killed *L. braziliensis* vaccine plus saponin; 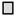); Leishmune^®^ (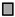); Leish-Tec^®^ (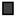). The *y*-axis represents the Stimulation Index that is expressed as the ratio of mean frequencies of the cells in the stimulated cultures over non-stimulated cultures (SLiA/CC ratio), where: (**A**) total lymphocytes proliferation; (**B**) specific CD4^+^ T lymphocytes proliferation; (**C**) specific CD8^+^ T lymphocytes proliferation. In the results of cytokine production (**D**) TNF-α and (**E**) IL-10, the *y*-axis represents the Stimulation Index that is expressed as the ratio of mean cytokine concentration (pg/mL) in the stimulated cultures over non-stimulated cultures (SLiA/CC ratio). Significant differences (*p* < 0.05) between the groups C, LBSap, Leishmune^®^, and Leish-Tec^®^ are indicated by connecting lines.

To explore the effects of the vaccination on in vitro cell proliferation, we measured lymphoproliferative activation of PBMCs after the third dose of vaccines (Figure 4). The analysis of the lymphoproliferative activity after the vaccination between the different groups revealed an increase (*p* < 0.05) in the proliferation index of the LBSap group in relation to the Control and Leish-Tec^®^ groups. In addition, a significant increase (*p* < 0.05) in the proliferation index in the Leishmune^®^ group was also observed in relation to the Control group (Figure 4A). The evaluation of the proliferation index of specific CD4^+^ T lymphocytes after vaccination revealed a significant increase (*p* < 0.05) in the LBSap group when compared to the Control, Leish-Tec^®^, and Leishmune^®^ groups (Figure 4B). Moreover, an increase (*p* < 0.05) in the specific CD8^+^ T lymphocytes in the LBSap group was also observed when compared to the Control and Leish-Tec^®^ groups (Figure 4C). In order to evaluate the cytokines produced by the PBMCs after SLiA stimuli in the culture supernatant, we evaluated the levels of TNF-α and IL-10 (Figure 4). We observed higher levels (*p* < 0.05) of TNF-α in the LBSap dogs after completing vaccination protocol when compared with the Control and Leish-Tec^®^ groups (Figure 4D). On the other hand, regarding the levels of IL-10 cytokine, no differences were observed between the groups (Figure 4E).

### 3.5. High Intracytoplasmic Synthesis of IFN-γ^+^ by CD8^+^ T Lymphocytes after In Vitro Antigen-Specific Stimulation Is a Marker of Immunogenicity Promoted by LBSap and Leish-Tec^®^ Vaccines

**Figure 5:**
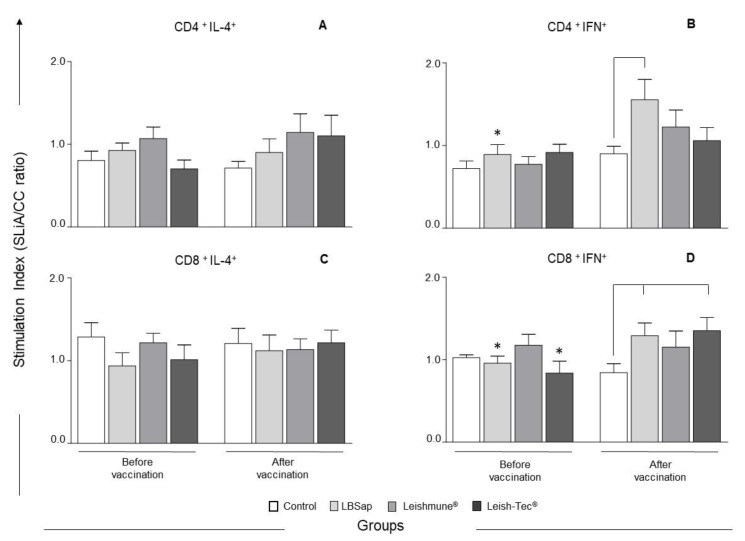
Profile of intracytoplasmatic cytokines in peripheral blood mononuclear cells (PBMCs) after stimulation with soluble *L. infantum* antigen (SLiA) in dogs submitted to different vaccination protocols: C (control; 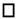); LBSap (killed *L. braziliensis* vaccine plus saponin; 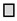); Leishmune^®^ (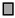); Leish-Tec^®^ (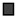). The *x*-axis displays the times of the assays (time before vaccination and time after vaccination) and the *y*-axis represents the Stimulation Index that is expressed as the ratio of mean frequencies of cytokines+ T cell subsets in the stimulated cultures over non-stimulated cultures (SLiA/CC ratio), wherein: (**A**) CD4^+^ IL-4^+^ T lymphocytes; (**B**) CD4^+^ IFN-γ^+^ T lymphocytes; (**C**) CD8^+^ IL-4^+^ T lymphocytes; (**D**) CD8^+^ IFN-γ^+^ T lymphocytes. Significant differences (*p* < 0.05) are shown by connecting lines—representing differences between the group C and the LBSap, Leishmune^®^, and Leish-Tec^®^ groups—and by the “*”—representing differences between time before vaccination and time after vaccination.

In order to evaluate the cytokine profile produced by T lymphocyte subsets, the intracytoplasmic synthesis of IFN-γ and IL-4 was assessed after antigen-specific stimulation (Figure 5). In relation to CD4^+^ T lymphocytes, we observed in the LBSap group a significant increase (*p* < 0.05) in the CD4^+^ IFN-γ^+^ T lymphocyte index after vaccination when compared to the index before vaccination. Furthermore, when assessing the differences between experimental groups after vaccination, only the LBSap group had a significant (*p* < 0.05) increase in the CD4^+^ IFN-γ^+^ T lymphocyte index when compared to the control group after the immunization protocol (Figure 5B). The LBSap group also showed a significant (*p* < 0.05) increase in the CD8^+^ IFN-γ^+^ T lymphocyte index when compared to the control group after vaccination (Figure 5D). In the LBSap group, there was also a significant (*p* < 0.05) increase in CD8^+^ IFN-γ^+^ T lymphocytes after vaccination when compared to the initial evaluation time prior to vaccination. In addition, the Leish-Tec^®^ group showed a significant (*p* < 0.05) increase in CD8^+^ IFN-γ^+^ T lymphocytes after vaccination in relation to the time before vaccination. Moreover, we observed a significant (*p* < 0.05) increase in the CD8^+^ IFN-γ^+^ T lymphocyte index in this group (Leish-Tec^®^) compared to the control group after vaccination (Figure 5D).

## 4. Discussion

Considering the necessity to invest and expand the development of anti-CVL vaccines that meet the prerequisites of the IN-31/2007, the detailed studies of immunobiological potential become fundamental to reach the purpose of a vaccine being used commercially and perhaps in the VL Surveillance and Control Program (VLSCP) in Brazil [29]. Therefore, the present study proposed to evaluate the safety, toxicity, and immunogenicity of the LBSap vaccine in a comparative study with the Leishmune^®^ and Leish-Tec^®^ vaccines in a phase I and II clinical trial in closed kennel.

The initial characterization of the vaccines evaluated in this study counted on the description of important parameters related to the biological safety conferred during the three immunizations. The safety and toxicity evaluations performed in this study did not identify clinical alterations that compromise the health of the animals immunized with the different vaccines, so that all products can be considered safe and innocuous. However, changes in the inoculum site with the LBSap and Leishmune^®^ groups were observed. These changes were discrete, since most animals only had the presence of small circumscribed, non-ulcerated nodules of firm consistency [33,38]. In some dogs of these groups, mild edema and palpation pain were also observed at the inoculation site of the vaccines. The presence of edema and pain at the site of the vaccine inoculum with Leishmune^®^ was also reported by Fernandes and colleagues [38], as well as described in the package leaflet of this vaccine, as possible adverse effects to vaccination. It is important to note that these effects were transient and regressed completely 72 h after vaccination [33]. Moreover, such adverse effects are strongly related to the adjuvant used in the vaccines, saponin, which was the only adjuvant employed for all the vaccine compositions tested here [25,38,39]. No hematological changes (leukogram and erythrogram) or biochemical changes (renal, hepatic function, and proteinogram) were observed at the end of the immunizations with the different vaccines as observed in other studies [30,40,41]. Some studies using saponin as an adjuvant report the presence of other adverse effects, which were not evidenced in our study, such as loss of hair at the inoculum site, alteration of coat color and hypotrichosis/alopecia, anorexia, apathy, prostration, vomiting, diarrhea, emesis, hypodipsia, hypophagia, salivation, tremors, breathing problems, angioedema, and, more rarely, allergic or anaphylactic reactions [25,39,42].

The production of humoral immune response is not directly related to protection derived from anti-*Leishmania* vaccination, and the CVL is often associated with a specific non-protective humoral response [43]. Some studies have proposed the analysis of Total IgG and its isotypes (IgG1 and IgG2) as complementary immunological biomarkers [13,22,44]. In addition, to attend the IN-31/2007 [29] for anti-CVL vaccine tests, which advocates for the identification of methods to distinguish between natural infection by *Leishmania infantum* and the immune response to the vaccine product, we performed the serum IgG reactivity evaluation by EIE^®^ and used the DPP^®^, both serological tests that are recommended and used by the Ministry of Health in the VLSCP in Brazil. Our results using DPP^®^ demonstrated that none of the dogs from the different vaccine groups in the study showed a positive test result, demonstrating that probably these vaccinated dogs do not react as false-positive in this test. The evaluation of total anti-*Leishmania* IgG antibodies (EIE^®^) showed an increase in the mean optical density of the LBSap and Leishmune^®^ groups above the positivity threshold. Previous studies [33] demonstrated, using the ELISA technique with SLiA, that vaccination with LBSap induces high levels of total IgG, IgG1, and IgG2 anti-*Leishmania*. In the case of LBSap vaccine which is composed of heterologous antigens of *L. braziliensis*, diseased animals may be distinguished from those vaccinated by serological methods, such as ELISA or immunochromatography, using specific antigens such as rk39, or by flow cytometry [45,46]. Similarly, seroconversion after vaccination of dogs with Leishmune^®^ has been reported, using SLiA through ELISA [41,47,48]. Our results in the Leish-Tec^®^ group differ from the serological results obtained by Fernandes and colleagues [38] in dogs immunized with Leish-Tec^®^ vaccine belonging to an endemic area to CVL. However, they are similar to the Leish-Tec^®^ vaccine phase I and II clinical trial in closed kennel where seroconversion of dogs was not observed during and after vaccination with the Leish-Tec^®^ prototype [30].

In this study, the immunophenotyping of canine leukocytes was performed by flow cytometry with the objective of identifying possible biomarkers related to the immunogenicity of the vaccine [13,18,49]. In the CVL natural history, Reis and colleagues [20] described that the decrease in the CD21^+^ B lymphocyte population would be related to increased parasite load in the bone marrow, presence of clinical signs of the disease, and prognosis of CVL severity. Thus, the increase in CD21^+^ B lymphocytes, characteristic of the group immunized with the Leishmune^®^ vaccine, appears to be a biomarker of resistance in CVL. In order to evaluate the profile of potential antigen-presenting cells induced during the vaccination process, as well as to expand the investigation of factors that could contribute to the activation of adaptive immunity, the number of circulating CD14^+^ monocytes was studied. In this context, an increase of CD14^+^ monocytes in the LBSap and Leishmune^®^ groups was observed at the end of the vaccine protocol. The increase in this population may be related to a possible migration of these cells to the immunization site. Vitoriano-Souza and colleagues [50] observed the possible augment of these cells involved in the late kinetic events of migration to the dermis and responsible for the cellular mechanisms involved in the local inflammatory process. In an attempt to evaluate activated T cells (CD25^+^) in vaccinated dogs, we observed an increase in circulating phenotype of CD4^+^ CD25^+^ T lymphocytes in the LBSap and Leishmune^®^ groups and an increase in the circulating percentage of CD8^+^ CD25^+^ T lymphocytes after the vaccine protocol in all groups. There are few studies in the literature that evaluated the regulatory T cells in the natural history of CVL [22,51], the results of Cortese and colleagues [52] revealed a reduced percentage of regulatory T CD4^+^, CD3^+^ and Foxp3^+^ subsets in both asymptomatic and symptomatic infected dogs in comparison with non-infected controls. The balance between regulatory and effector T cells is important for maintaining appropriately effective immune responses [53].

To determine whether the vaccines would be able to activate PBMCs under in vitro antigenic stimulation with SLiA, we measured the stimulation index in cells derived from immunized dogs. Analysis of lymphoproliferative activity after the three vaccine doses showed an increased proliferation index in the cultures stimulated with SLiA in the LBSap and Leishmune^®^ groups, indicating the recognition of antigens related to the etiological agent of CVL. Only the LBSap vaccine was able to produce an increase in the specific proliferation index of the subpopulations of T lymphocytes (CD4^+^ and CD8^+^). Similar to our findings, Fiuza and colleagues [41] also did not observe an increase in the antigen-specific proliferation of T lymphocytes (CD4^+^ and CD8^+^) of dogs immunized with the Leishmune^®^ vaccine. The specific proliferation of CD4^+^ T lymphocytes against SLiA in the LBSap group is an important biomarker of protective immunogenicity in CVL [13]. Ramos and colleagues [54] observed that only vaccinated dogs, which remained asymptomatic after experimental infection, presented increased CD4^+^ T lymphocytes after in vitro culture of lymph node cells, confirming the importance of these cells in the activation of an immune response to control the parasite. Similar to our results, Giunchetti and colleagues [33,34] also observed an increase in the frequency of antigen-specific CD8^+^ T lymphocytes in the LBSap and LBSapSal groups. Reis and colleagues [21] showed a higher number of CD8^+^ T lymphocytes in the spleen of asymptomatic dogs after in vitro stimulation with SLiA, evidencing the importance of these cells in mechanisms related to resistance to *L. infantum* infection in dogs. In addition, in human beings and dogs, asymptomatic infection by *L. infantum* has been associated with the expansion of *Leishmania*-specific CD8^+^ T lymphocytes [13,55]. Associated with in vitro lymphoproliferative response, we observed an increased production of TNF-α in the LBSap group with no differences in the levels of IL-10 production between the different vaccine groups. These results also confirm the effect promoted by LBSap vaccination on activating a pro-inflammatory cell immune response consistent with the development of a protective immunity against the disease [56,57].

Considering the combined analysis of T cell subpopulations and in vitro cytokine production after specific stimulation with SLiA, both CD4^+^ and CD8^+^ T cells are relevant sources of IFN-γ after vaccination with the LBSap vaccine. On the other hand, these cells did not secrete IL-4 differently between the tested vaccine groups. It is known that in VL, whether in human or canine disease, the proinflammatory cytokine IFN-γ stimulates increased cytotoxic activity of NK cells. The development of Th1 cells promotes the activation of lymphocytes and the microbicidal activity of macrophages against the parasites through the production of hydrogen peroxide and/or nitric oxide (NO) [15,18]. Resende and colleagues [57] observed, in PBMC of dogs immunized with LBSap vaccine, the production of IL-12, IFN-γ, and nitric oxide in supernatant of cultures stimulated with SLiA, after the vaccine protocol as well as after the experimental challenge. Different from our findings with the LBSap vaccine, immunization with crude antigens of *L. amazonensis* associated with Bacille Calmette-Guerin led to a mixed immune response profile, characterized by the simultaneous increase of IFN-γ and IL-4 in T lymphocytes stimulated in vitro with SLiA [40,58]. Similar to our findings, Fiuza and colleagues [41] also found no difference in the frequency of IL-4 or IFN-γ producing (CD4^+^ and CD8^+^) lymphocytes after immunization of dogs with the Leishmune^®^. Our results in the Leish-Tec^®^ group also demonstrated the ability of this immunobiological to promote increased CD8^+^ T lymphocytes producing IFN-γ after immunizations. In fact, PBMC from dogs immunized with the Leish-Tec^®^ vaccine demonstrated to be able to increase the production of the cytokine IFN-γ in supernatant of cultures stimulated with the rA2 antigen after the immunizations and after the experimental challenge [30]. These and other studies have shown that IFN-γ is a high-quality biomarker in the identification of an immunogenic and protective vaccine against *Leishmania* infection [13,15,30,40,41,57,58].

## 5. Conclusions

This is the first phase I and II clinical trial study that compared LBSap with two commercial vaccines. The results obtained in this study showed that the immunobiological LBSap, as well as the commercial Leishmune^®^ and Leish-Tec^®^ vaccines, did not induce adverse changes that would contraindicate its use. In addition, the results of the assessment of the immunogenicity aspects obtained with the LBSap, Leishmune^®^, and Leish-Tec^®^ vaccines indicate the establishment of immunoprotective mechanisms potentially capable of acting against infection by *L. infantum* and consequently in the prevention of CVL. Thus, our results showed that the LBSap vaccine presented several biomarkers related to protection against leishmaniasis and could be considered a vaccine candidate to proceed in a randomized double-blind phase III clinical trial.

## Supplementary Materials

The following are available online at https://www.mdpi.com/2076-393X/8/4/690/s1, Table S1: Evaluation of Safety and Toxicity in control dogs and dogs submitted to different vaccine protocols, Table S2: Leukogram of control dogs and dogs submitted to different vaccine protocols, Table S3: Erythrogram of control dogs and dogs submitted to different vaccine protocols, Table S4: Biochemical analyses of hepatic function of control dogs and dogs submitted to different vaccine protocols, Table S5: Biochemical analyses of the proteinogram and renal function of control dogs and of dogs submitted to different vaccine protocols.
