# Supplementary material for: Phase I and II Clinical Trial Comparing the LBSap, Leishmune®, and Leish-Tec® Vaccines against Canine Visceral Leishmaniasis
Source: Vaccines (Basel). 2020 Nov 17;8(4):690. doi: 10.3390/vaccines8040690 (PMC7712644; doi:10.3390/vaccines8040690)
Supplement: Supplementary file 1 [file vaccines-08-00690-s001.pdf]

Table S1. Evaluation of Safety and Toxicity in control dogs and dogs submitted to different vaccine protocols.

Groups	Time	Weight loss	Fever	Edema/Nodule	Local pain
	24–72h after 1ºdose				
Control		-	-	-	-
LBSap		-	1	3	-
Leishmune		-	-	2	-
Leish-Tec		-	-	-	-
	24–72h after 2ºdose				
Control		-	-	-	-
LBSap		-	3	2	-
Leishmune		-	2	2	-
Leish-Tec		-	-	-	-
	24-72h after 3ºdose				
Control		-	-	-	-
LBSap		-	4	7	1
Leishmune		-	-	5	1
Leish-Tec		-	-	-	-

Number of dogs in each of the vaccine groups that presented some of the physiological and / or behavioral changes evaluated, such as: Weight loss; Fever (rectal temperature above 39.5 °C); changes in the site of immunizations (nodule or edema; local pain); for 72 hours after each of the three vaccinations in the control groups (C); saponin-associated *L. braziliensis* vaccine (LBSap); Leishmune® vaccine; Leish-Tec® vaccine; Evaluation times: 24–72h 1st dose = twenty four to seventy-two hours after the first dose of immunization; 24–72h 2nd dose = twenty four to seventy-two hours after the second immunization dose; 24–72h 3rd dose = twenty four to seventy-two hours after the third dose of immunization. The number of dogs in each group was 7. The " - " represents that there was no change in that group.

Table S2. Leukogram of control dogs and dogs submitted to different vaccine protocols.

Groups	Times	Global Leukocytes	Lymphocytes	Monocytes	Neutrophils	Eosinophils
Reference values		7408–14440 (mm^3)	2299–5119 (mm^3)	147–601 (mm^3)	3839–9379 (mm^3)	150–710 (mm^3)
	T0					
Control [a]		10,660 ± 777	3359 ± 242	323 ± 136	6191 ± 1344	424 ± 196
LBSap [b]		10,414 ± 1389 [T1]	3537 ± 564	354 ± 125	6158 ± 1132 [T1]	230 ± 221
Leishmune [c]		12,020 ± 2367	3691 ± 704	376 ± 151	7160 ± 1844	493 ± 336
Leish-Tec [d]		11,625 ± 850	3961 ± 710	445 ± 200	7035 ± 1511	467 ± 387
	T1					
Control [a]		10,057 ± 1756	3320 ± 676	358 ± 64	5974 ± 1228	516 ± 237
LBSap [b]		12,371 ± 1699 [a]	3003 ± 351	364 ± 82	8559 ± 1963 [a]	445 ± 292
Leishmune [c]		12,533 ± 2386	3336 ± 794	366 ± 218	7714 ± 2212	806 ± 343
Leish-Tec [d]		12,150 ± 2545	3467 ± 853	330 ± 54	7768 ± 1721	585 ± 351

Absolute values (mean ± standard deviation) of the leukogram of dogs submitted to different vaccine protocols: control (C); *L. braziliensis* vaccine plus saponin (LBSap); Leishmune® vaccine (LM); Leish-Tec® vaccine (LT); Evaluation times: T0 = before the first immunization; T1 = 15 days after the third immunization; The significant differences ($p < 0.05$) are represented by the letters a, b, c, and d related to groups C, LBSap, LM, LT, respectively. The significant differences ($p < 0.05$) in the longitudinal evaluation of each leukogram parameter are represented by the abbreviations T0 and T1.

Table S3. Erythrogram of control dogs and dogs submitted to different vaccine protocols.

Groups	Times	Red Blood Cells	Hemoglobin	Hematocrit	Platelets
Reference values		5.42–8.38 (10^6/mm^3)	13.2–21.2 (g/dL)	33.7–57.3 (%)	171–384 (10^3/mm^3)
	T0				
Control [a]		7.20 ± 0.69	17.9 ± 2.1	50.2 ± 5.5	282 ± 71
LBSap [b]		6.81 ± 0.58	17.5 ± 1.5	44.4 ± 6.5	268 ± 74
Leishmune [c]		7.25 ± 1.06	18.4 ± 2.1	46.7 ± 6.4	245 ± 108
Leish-Tec [d]		7.22 ± 0.32	18.3 ± 1.1	48.6 ± 4.7	237 ± 37
	T1				
Control [a]		7.15 ± 0.63	18.4 ± 2.3	44.9 ± 5.2	296 ± 63
LBSap [b]		7.13 ± 0.76	18.4 ± 2.1	44.9 ± 5.1	306 ± 93
Leishmune [c]		7.77 ± 0.67	19.7 ± 1.4	48.5 ± 4.0	287 ± 46
Leish-Tec [d]		7.16 ± 0.40	18.4 ± 1.0	44.5 ± 3.4	344 ± 31

Absolute values (mean ± standard deviation) of the erythrogram (total red blood cells, hemoglobin, hematocrit and platelets) of dogs submitted to different vaccine protocols: control (C); *L. braziliensis* plus saponin (LBSap); Leishmune® vaccine (LM); Leish-Tec® vaccine (LT); Evaluation times: T0 = before the first immunization; T1 = 15 days after the third immunization. The significant differences ($p < 0.05$) are represented by the letters a, b, c, and d related to groups C, LBSap, LM, LT, respectively.

Table S4. Biochemical analyses of hepatic function of control dogs and dogs submitted to different vaccine protocols.

Groups	Times	TGO/AST	TGP/ALT	Gama-GT	Alkaline phosphatase	Total Bilirubin
Reference values		26.1–48.1 (U/L)	31.2–63.2 (U/L)	0.6–7.8 (U/L)	11.8–51.0 (U/L)	0.05–0.69 (mg/dL)
	T0					
Control [a]		38.7 ± 5.6	49.4 ± 5.9	3.7 ± 1.9	28.0 ± 9.9	0.42 ± 0.17
LBSap [b]		34.7 ± 5.8	45.3 ± 6.3	4.9 ± 1.8	28.6 ± 8.2	0.44 ± 0.13
Leishmune [c]		37.4 ± 3.4	48.4 ± 8.8	4.8 ± 2.1	24.3 ± 5.7	0.49 ± 0.15
Leish-Tec [d]		38.2 ± 5.1	49.0 ± 10.6	5.1 ± 1.6	32.0 ± 9.5	0.28 ± 0.08
	T1					
Control [a]		38.3 ± 4.1	44.0 ± 10.1	5.9 ± 0.9	36.0 ± 8.2	0.27 ± 0.16
LBSap [b]		38.0 ± 4.1	44.3 ± 10.2	6.0 ± 1.7	42.3 ± 5.2	0.26 ± 0.14
Leishmune [c]		41.0 ± 4.9	52.2 ± 11.2	6.8 ± 1.3	36.8 ± 6.8	0.39 ± 0.21
Leish-Tec [d]		40.1 ± 4.6	49.8 ± 7.5	4.5 ± 2.4	30.0 ± 5.4	0.29 ± 0.18

Absolute values (mean ± standard deviation) of hepatic function (TGO / AST, TGP / ALT, Gamma-GT, alkaline phosphatase, total bilirubin) of dogs submitted to different vaccine protocols: control (C); *L. braziliensis* plus saponin (LBSap); Leishmune® vaccine (LM); Leish-Tec® vaccine (LT); Evaluation times: T0 = before the first immunization, T1 = 15 days after the third immunization. The significant differences ($p < 0.05$) are represented by the letters a, b, c, and d related to groups C, LBSap, LM, LT, respectively.

Table S5. Biochemical analyses of the proteinogram and renal function of control dogs and of dogs submitted to different vaccine protocols.

Groups	Times	Total Protein	Albumin	Globulin	Ratio A/G	Urea	Creatinine
Reference values		6.4–8.9 (g/dL)	1.5–6.7 (g/dL)	0.7–6.3 (g/dL)	0.05–3.25	28.0–40.1 (mg/dL)	0.6–1.5 (mg/dL)
	T0						
**Control [a]**		7.5 ± 0.6	4.9 ± 0.6	2.6 ± 0.9	2.2 ± 1.1	35.6 ± 1.0	1.2 ± 0.3
**LBSap [b]**		7.3 ± 0.6	4.6 ± 0.5	2.7 ± 0.8	1.9 ± 0.7	36.2 ± 1.0	1.2 ± 0.2
**Leishmune [c]**		7.8 ± 0.6	5.0 ± 1.0	2.7 ± 0.8	2.1 ± 1.1	36.7 ± 1.5	1.0 ± 0.1
**Leish-Tec [d]**		7.5 ± 0.8	4.7 ± 0.4	2.8 ± 1.0	1.9 ± 0.9	36.9 ± 1.5	1.1 ± 0.2
	T1						
**Control [a]**		6.7 ± 1.9	2.4 ± 0.6	3.5 ± 1.3	0.7 ± 0.4	29.5 ± 1.6	1.1 ± 0.1

**LBSap** [b]	6.7 ± 1.9	2.3 ± 0.9	4.1 ± 1.0	0.5 ± 0.2	32.1 ± 3.1	1.0 ± 0.1
**Leishmune** [c]	6.7 ± 1.0	2.5 ± 0.9	4.2 ± 0.5	0.6 ± 0.2	30.9 ± 5.9	1.0 ± 0.1
**Leish-Tec** [d]	6.8 ± 1.2	2.2 ± 0.7	5.1 ± 1.1	0.4 ± 0.1	30.1 ± 1.5	1.0 ± 0.1

Absolute values (mean ± standard deviation) of the proteinogram (total protein, albumin, globulin and albumin / globulin - A / G ratio) and renal function (urea and creatinine) of dogs submitted to different vaccine protocols: control (C); *L. braziliensis* plus saponin (LBSap); Leishmune® vaccine (LM); Leish-Tec® vaccine (LT). Evaluation times: T0 = before the first immunization, T1 = 15 days after the third immunization. The significant differences ($p < 0.05$) are represented by the letters a, b, c, and d related to groups C, LBSap, LM, LT, respectively.
